# pH-Responsive Polyethylene Glycol Monomethyl Ether-ε-Polylysine-G-Poly (Lactic Acid)-Based Nanoparticles as Protein Delivery Systems

**DOI:** 10.1371/journal.pone.0159296

**Published:** 2016-07-28

**Authors:** Huiqin Liu, Yijia Li, Rui Yang, Xiujun Gao, Guoguang Ying

**Affiliations:** 1 Laboratory of Cancer Cell Biology, Tianjin Key Laboratory of Cancer Prevention and Therapy, National Clinical Research Center for Cancer, Tianjin Medical University Cancer Institute and Hospital, Tianjin, 300060, China; 2 Institute of Biomedical Engineering, Tianjin Medical University, Tianjin, 300070, China; 3 Research Center of Basic Medical Sciences, Tianjin Medical University, Tianjin, 300070, China; Brandeis University, UNITED STATES

## Abstract

The application of poly(lactic acid) for sustained protein delivery is restricted by the harsh pH inside carriers. In this study, we synthesized a pH-responsive comb-shaped block copolymer, polyethylene glycol monomethyl ether-ε-polylysine-*g*-poly (lactic acid) (PEP)to deliver protein (bovine serum albumin (BSA)). The PEP nanoparticles could automatically adjust the internal pH to a milder level, as shown by the quantitative ratio metric results. The circular dichroism spectra showed that proteins from the PEP nanoparticles were more stable than those from poly(lactic acid) nanoparticles. PEP nanoparticles could achieve sustained BSA release in both *in vitro* and *in vivo* experiments. Cytotoxicity results in HL-7702 cells suggested good cell compatibility of PEP carriers. Acute toxicity results showed that the PEP nanoparticles induced no toxic response in Kunming mice. Thus, PEP nanoparticles hold potential as efficient carriers for sustained protein release.

## Introduction

From 1980s, FDA has approved above 400 biopharmaceuticals. Nowadays, biopharmaceuticals are becoming the leading therapeutics owing to their clinical and commercial success. Peptides and proteins are of a large class of biopharmaceuticals under investigation [1]. Poly(lactic acid) (PLA) and poly(lactic-co-glycolic acid) (PLGA) are approved for human use as sustained release drug delivery systems [2]. However, application of these compounds is limited by several protein-damaging factors [3, 4]. For example, the acidic microclimate inside these types of carriers negatively affects encapsulated proteins [5, 6].We previously designed PLGA derivatives to neutralize the acidic microenvironment in PLGA. However, drawbacks caused by the acidic microenvironment in PLA carriers persist.

PLA-based drug delivery systems exhibit certain unique properties that may be advantageous under various physiological conditions, compared with PLGA-based systems. For example, PLA exhibits higher hydrophobicity than PLGA, which leads to different degradation characteristics. The degradation and erosion of PLA are also more time consuming than those of PLGA, so PLA can be effectively used for prolonged drug release [7]. The release properties, especially the release time, of carriers derived from PLA derivatives can be accurately adjusted to fulfill special requirements. Such tailorable features are always desirable in biomedical applications, which can require release kinetics ranging from several days to as long as 6 months.

We have designed a novel strategy to improve protein stability within PLA-based nanoparticles (NPs). Hydrophilic or cationic materials were introduced to neutralize the acidic conditions that accompany the hydrolysis of PLA, thereby stabilizing several encapsulated proteins [8, 9]. This study was performed to prepare comb-shaped amphiphilic polyethylene glycol monomethyl ether-ε-polylysine-g-poly(lactic acid) [CH3-PEG-EPL-g-PLA;PEP] to form pH-responsive PLA-based NPs for protein delivery.

## Materials and Methods

### Materials

PLA (molecular weight (M_W_): 8.0 kDa) was obtained from Jinan Daigang Biomaterial Co., Ltd. (Jinan, China). ε-polylysine (EPL) (M_W_, 4.08 kDa) was obtained from Fanqing Biochemical Co., Ltd. (Peking, China). Methoxypolyethylene glycol amine (CH3-PEG-NH_2_; M_w_, 1.1, 2.1, 5.1, and 10.1 kDa) was obtained from Shanghai Rebone Biomaterials Co., Ltd. (Shanghai, China). The fluorescent pH-sensitive dye, SNARF-1(R) dextran (M_W_,10 kDa), was obtained from Shanghai Haoran Bio Technologies Co., Ltd. 1-Ethyl-(3-3-dimethylaminopropyl) carbodiimide hydrochloride (EDC∙HCl), 1-hydroxybenzotrizole (HOBt), fluorescein isothiocyanate (FITC), 3-(4,5-dimethylthiazol-2-yl)-2,5-diphenyltetrazoliumbromide (MTT), di-tert-butyl dicarbonate (BOC), trifluoroacetic acid, and hematoxylin and eosin (H&E) were obtained from Sigma. Propofol was purchased from Tianjin Lianxing Bio Technologies Co., Ltd. Bovine serum albumin (BSA; M_W_, 67 kDa) and a BCA protein assay kit were obtained from Junyao Weiye Biochemical Co., Ltd. (Peking, China). Micropore filters and ultrafiltration tubes were obtained from Tianjin Lianxing Bio Technologies Co., Ltd. The dialysis bags were obtained from Shanghai Green Bird Sci. and Tech. Co., Ltd. Other reagents were obtained from business companies. All animals were purchased from Tianjin Aoyide Experimental Animal Technology Co., Ltd.

### PEP Preparation & Characterization

We synthesized the functional PEP copolymers via classical amide bond forming reactions as described previously [10]. For BOC-protected EPL (BOCEPL), the BOC groups protected the primary amines of EPL. BOCEPL-PEG-CH3 was obtained using EDC∙HCl and HOBt to link CH3-PEG-NH_2_ and BOCEPL. EPL-PEG-CH3 was obtained by BOC deprotection of BOCEPL-PEG-CH3 using trifluoroacetic acid. The coupling reaction of PLA with EPL-PEG-CH3 was performed using HOBt and EDC∙HCl to yield the final product CH3-PEG-EPL-g-PLA (PEP). ^1^H NMR spectroscopy (Bruker Avance III 400) was used to determine the copolymer structure and the result was shown in S1 Fig.

The polydispersity and M_w_ of PEP were determined by gel permeation chromatography (GPC) [11] using a Waters system (Waters, USA) that included a Waters 600 E pump. The flow rate of the pump was 1.0 mL/min, while the columns (PL-M-B) were kept under 50°C. To prepare samples, we dissolved the material in DMF (10 mL) and then filtered it (0.22 μm). We measured the M_w_ of PEP using polystyrene standards.

### NP Structure, Zeta Potential, and Size

We employed nanoprecipitation to prepare NPs [10]. DMF (10 mL) was used to dissolve PEP (concentration: 10 mg/mL). Double distilled water (DDW) (100 mL) was used to dissolve BSA (concentration: 0.4 mg/mL). Then, the PEP solution was added to the aqueous phase drop wise, and the mixture was stirred for 15 min. The mixtures were dialyzed against DDW (5,000 mL) for 48 h at 4°C to obtain the final NP solution. The abovementioned method was also used to produce blank NPs in the absence of BSA. Then, the obtained NP solution was freeze-dried and stored under 4°C.

We performed transmission electron microscopy (TEM) using a Hitachi HT7700 instrument. One NP drop was put onto a copper grid of 200 meshes to equilibrate. We removed the water from the copper grid after NPs deposition using a filter paper before loading into the microscope.

We used Zetasizer 3000 HS (Brookhaven) to measure the zeta potential of the NP sat 37°C. The NP size and polydispersity were detected through dynamic light scattering (Brookhaven, INNDVO300/BI900AT). X-ray photoelectron spectroscopy (XPS) was used to examine the chemical composition of NP shaving PEG of different M_w_.

Lyophilized NPs were subsequently re-suspended in 1.0 mL D_2_O for ^1^H NMR analysis (determination of surface composition of NPs).

### NP Water Absorption and Degradation

We measured the water absorption of the PEP NPs using previously described procedures [10]. Approximately 20mg of dried sample was added to phosphate buffered saline (PBS, 0.1 M, pH 7.4) at37°C. The product obtained after 48 h was cautiously blotted off between tissue paper sheets. The sheets were weighed with an electronic microbalance (AE 240, Mettler, Switzerland) (accuracy: ±0.01 mg). We computed the proportion of water absorption according to the following formula:

$$Water\;uptake\;\left(\%\right)\;=\;\left(W_{w}-W_{d}\right)/W_{d}\times 100\%$$ (1)

where *W*_*w*_ is the swollen sample weight, and *W*_*d*_ represents original weight. The experiment was repeated three times, and the data are represented as the mean values ± the standard error.

The residual weight was computed as follows:

$$Residual\;mass\;\left(\%\right)\;=\;W_{c}/W_{i}\times 100\%$$ (2)

where *Wi* represents original sample weight and *Wc* represents constant weight after drying at different time points.

### BSA Release and Kinetics

Approximately 20 mg of BSA-loaded NPs was soaked in 5 mL of PBS (0.1 M, pH 7.4) and maintained at 37°C with horizontal shaking. Then, 100μL aliquots of the sample were transferred into an ultrafiltration tube at different time points and centrifuged at 8,000 rpm for 15 min. We then removed the liquid and resuspended the sample with 100 μL of PBS.A BCA protein assay was employed to detect BSA in the supernatant after centrifugation. We examined the samples in triplicate for each test.

The mechanism of PEP NP protein release was evaluated using two models, namely, the Ritger–Peppas and biexponential equations.

Model 1 was established using the Ritger–Peppas equation:

$$M_{t}/M_{\infty }=K_{RP}t^{n}\left(M_{t}/M_{\infty }\leq 60\%\right)$$ (3)

where *M*_*t*_*/M*_*∞*_ denotes the ratio of released drug, *K*_*RP*_ represents the geometric shapes of the carrier, *t* indicates the release time, and *n* denotes the release exponent relevant to the mechanism of drug release. Fickian diffusion is observed when *n*≤ 0.43 for drug release from spherical matrices. When the value of *n* was between 0.43 and 0.85, the mechanism of drug release is described as non-Fickian (anomalous) [12].

The following formula describes Model 2, which is suitable for the release data in this study:

$$M_{t}/M_{\infty }=\;a_{1}\left[1-exp\left(-k_{1}t\right)\right)+a_{2}\left(1-exp\left(-k_{2}t\right)\right]$$ (4)

where *k*_*1*_ and *k*_*2*_represent the first-order rate constants, and *a*_*1*_ and *a*_*2*_ are the percentages of the BSA on the outer layer and inside the NPs, respectively[13].

We used adjusted coefficient of determination (R^2^_adjusted_) to identify whether the model could properly described the release data. The adjusted coefficient of determination was considered to best explain the data.

$$R^{2}{}_{adjusted}=1-\left(n-1\right)\left(1-R^{2}\right)/\left(n-p\right)$$ (5)

### Biocompatibility of PEP NPs

MTT was employed to measure the viability oftheHL-7702 cells. The viability of each cell was computed as below:

$$Cell\;viability\;\left(\%\right)\;=\;\left(Abs_{test}/Abs_{control}\right)\times 100\%$$ (6)

where *Abs*_*test*_ represents the amount of formazan in the cells with NP administration, and *Abs*_*control*_ represents the amount of formazan in the controlled cells (with no NP treatment).

We assessed acute toxicity of PEP NPs and EPL using male and female Kunming mice weighing 20–25 g each (7–8 weeks) after tail vein injection according to our previous study [10].

We then conducted an exploratory assay to investigate the dose range for determining the LD50 of PEP and PLA NPs. The exploratory assay was performed in 30 mice. We randomly assigned the mice into three groups (labeled as A, B, and C), each of which contained 10 animals. The doses of the PEP and PLA NP solutions, which were diluted in a sodium chloride (NaCl) solution (0.9%), were 12 mg/mL and 3 mg/mL, respectively. These doses were the highest concentrations of the PEP and PLA NPs prepared with nanoprecipitation. The experimental groups (A and B) were treated with continuous intravenous administrations of PEP and PLA NP solutions for 12 h (0.3 mL/h), respectively. The control mice were treated with continuous administration of a NaCl solution (0.9%) intravenously for 12 h (0.3 mL/h). We observed the mice for 14 days with an interval of six hours to evaluate their mortality and physiological behaviors. No deaths were observed in the animals administered with PEP and PLA NPs. Thus, theLD50 of PEP and PLA NPs could not be determined.

The aforementioned method was also used to detect the acute toxicity of EPL. However, the continuous intravenous treatment was replaced with a single administration of EPL solution intravenously. We randomly assigned the mice into seven groups (labeled as A, B, C, D, E, F, and G), each of which contained 10 animals. EPL was diluted in a NaCl solution (0.9%), with doses of 450.0, 383.0, 321.9, 270.5, 227.3, and 191.0 mg/kg for the experimental groups (A, B, C, D, E, and F). The animals in the experimental groups received a one-time administration of EPL solution (0.2 mL) intravenously. The control mice were treated with a one-time administration of 0.2 mL of a NaCl solution (0.9%) intravenously.

The animals were sacrificed on the 14^th^ day following intravenous injection in the acute experiment. Kunming mice were euthanized with carbon dioxide. Carbon dioxide was introduced into the box slowly. When the concentration of carbon dioxide reached to 99.9%, carbon dioxide was introduced continually into the box for 10 minutes. Then, the carcasses were sterilized by 75% ethanol and were transferred to the laminar flow bench for dissection. Three internal organs, namely, the liver, kidney, and spleen, were acquired from each animal. The tissues were immediately fixed in 10% formalin and dehydrated for paraffin wax embedding. We sectioned each embedded specimen and stained them with H&E. The images were visualized with an Olympus IX71 microscope.

All the animals received humane care in strict accordance with the Regulations for the Administration of Affairs concerning Experimental Animals (Tianjin, revised in June 2004), which conforms to the Guide for the Care and Use of Laboratory Animals published by the US National Institutes of Health (NIH Publication no. 85–23, revised 1996).

### Mapping of Microclimate pH within PEP and PLA NPs

We used a ratio metric method to map the pH within NPs according to previous descriptions [10].We used anAr/He laser to excite the encapsulated fluorescent dye (SNARF-1 dextran) at 488 nm and acquired the data I_640_/I_580_,the relationship of the ratio (I_640_/I_580_) and pH was obtained from a standard curve. All detection was performed with a 63× oil immersion objective.

### Secondary Structural Stability of Released Protein

BSA-loaded NPs (100 mg) were dispersed in 2 mL of sterilized DDW. The solution was transferred into 5 mL ultrafiltration tubes (100 kDa cutoff) at specified time intervals and centrifuged at 4°C (8,000 rpm, 30 min). We employed a BCA protein colorimetric assay to evaluate the protein release concentration. The NPs were re-dissolved in 2 mL of sterilized DDW for continued release. The stability of the released protein (with the same concentration as that in the DDW) from the NPs was assessed by measuring the CD spectra on a Jasco-715 Spectropolarimeter (JASCO, Tokyo, Japan) at 37°C with constant nitrogen gas flow. The spectra showed the average of 8–20 scans, and CD intensities were represented as mdeg.

### Distribution and Pharmacokinetics of NPs

The tests on the carriers were performed using 50 Kunming mice (25 male and 25 female), with a weight of 20–25 g each (7–8 weeks). We randomly assigned the mice into five groups (labeled as A, B, C, D, and E), with 10 animals in each group.

Testing solutions containing BSA-FITC-loaded PEP NPs (group A), blank PEP NPs as the control of the PEP NPs loaded with BSA-FITC (group B), BSA-FITC-loaded PLA NPs (group C), blank PLA NPs (as the control of the BSA-FITC-loaded PLA NPs; group D), and free BSA-FITC (group E) were used in the *in vivo* experiments. The experimental groups were given one-time administrations of the testing solutions (0.4 mL) intravenously. Simultaneously, the control group (group E) animals were injected with 0.4 mL of a NaCl solution (0.9%).

We anaesthetized the mice by peritoneal injection of propofol (15 mg/kg). Blood was drawn from abdominal aorta, 0.2 mL each time. Blood was drawn from each animal for *in vivo* BSA-FITC examination. The blood solution was mixed with heparin was centrifuged at 5,000 rpm for 10 min at 25°C. Afterward, 100 μL of plasma samples were acquired, diluted to 3 mL with 0.9% NaCl solution, and evaluated by fluorescence spectroscopy (Ls55, Perkin Elmer).

The amounts of BSA-FITC that accumulated in the liver, kidney, and spleen tissues were also detected. The animals were euthanized (the method was shown in “Biocompatibility of PEP NPs”), and their livers, kidneys, and spleens were collected at different time points (1 hour, 2 hours, 4 hours, 8 hours, 16 hours, 32 hours, 64 hours, 128 hours, 256 hours and 384 hours, respectively). After three washes with physiological saline, the organs were blotted off cautiously between tissue paper sheets. The sheets were weighed on an electronic microbalance and transferred to homogenizers to obtain the tissue homogenates. Subsequently, the homogenates were diluted with 1 mL of physiological saline, transferred into a 15 mL centrifuge tube, and then centrifuged at 8,000 rpm for 30 min at 4°C. The supernatants were collected, and precipitates were washed twice with 1 mL of physiological saline. The supernatants were combined and diluted to 5 mL with physiological saline. Impurities were removed with 0.22 μL micropore filters. The BSA-FITC concentrations were measured by fluorescence spectroscopy.

The amounts of BSA-FITC in the blood and organs (livers, kidneys, and spleens) were determined using a linear standard calibration curve.

The AUC_t0-t_ value was measured by assessing the area under BSA-FITC curves between *t*_*0*_ and *t* according to the trapezoidal rule [14].

### Statistical Analysis

All data are expressed as the mean ± standard deviation. Significant differences were assessed with a*t-*test and are significant when *P*<0.05.

## Results and Discussion

### PEP Preparation and Characterization

**Fig 1:**
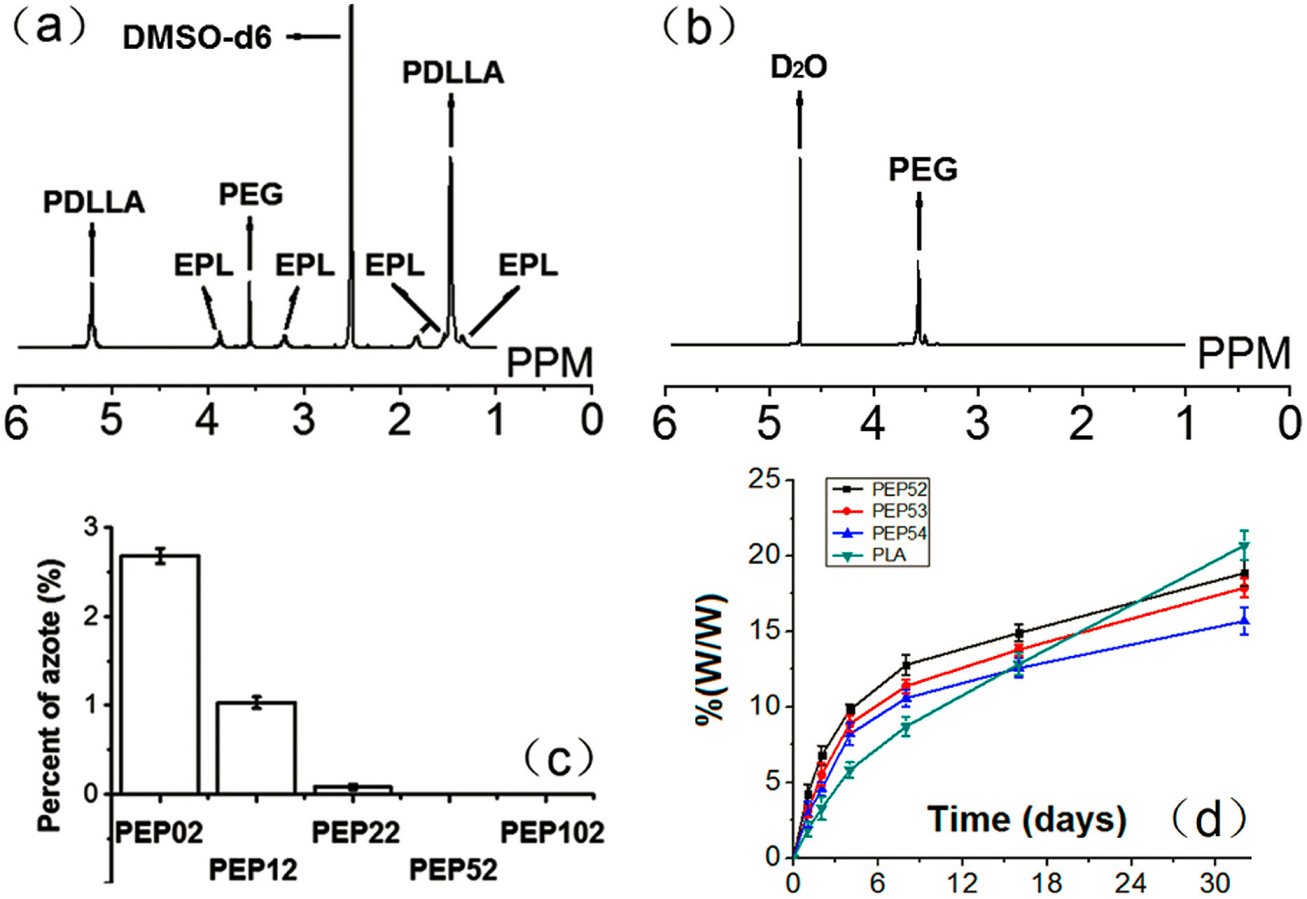
Structure of the amphiphilic comb-shaped copolymer PEP and biodegradation properties of PEP nanoparticles. (a) 1H-NMR spectrum of PEP in DMSO-d6; (b) 1H-NMR spectrum of PEP NPs in D2O; (c) chemical composition percentages of azote on the surface of different NPs; (d) Biodegradation properties of PEP and PLA NPs in PBS (0.1 M, pH 7.4) at 37°C.

The number of PLA chains grafted onto EPL-PEG-CH3 increased with an increase in the feed ratio of PLA to EPL-PEG-CH3 (Table 1). This indicates a steric effect between free PLA and PLA-g-EPL-PEG-CH3. For similar reaction times, the degree of PLA grafting was most affected by the feed ratio of PLA to EPL-PEG-CH_3_. The^1^H NMR spectrum of PEP is displayed in Fig 1a. The peak at δ 2.5ppm was assigned to residual protonated DMSO (DMSO-d6). The peak at δ 3.5 was attributed to the methylene protons of PEG. The peaks at δ 1.3–1.8, 3.3, and 3.9 were attributed to C_δ_H_2_-C_γ_H_2_-C_β_H_2_, C_ε_H_2_, and C_α_H of EPL, respectively. The peaks at δ 5.2 and 1.4 were attributed to the methine and methyl protons of PLA, respectively.

**Table 1:** Materials prepared with reagents of different molar ratios.

Materials	PEG (kDa)	PEG-EPL/PDLLA (mol/mol)	^Δ^GD	M_n_ (kDa)	PDI	Yield (%)
PEP02	—	1:3.1	2	20.4	1.1	78
PEP12	1.1	1:3.9	2	21.3	1.2	75
PEP22	2.1	1:4.1	2	22.5	1.2	75
PEP52	5.1	1:4.5	2	25.1	1.2	72
PEP53	5.1	1:5.7	3	33.3	1.3	66
PEP54	5.1	1:8.4	4	41.7	1.5	49
PEP102	10.1	1:5.2	2	30.2	1.3	68
PDLLA	—	—	—	8.0	1.1	—

^Δ^GD represents PDLLA graft degrees onto PEG-EPL. The PDLLA used had the same type, with a molecular weight of 8 kDa. M_n_ and PDI (M_w_/M_n_) were measured via GPC, as described above.

GPC was performed to confirm the M_w_ and structures of the copolymers (Table 1). The product PEP52, comprised of two PLA chains grafted onto one EPL-PEG-CH_3_ molecule, was obtained using a feed ratio of 1:4.5 (EPL-PEG-CH_3_ to PLA). The M_w_ of EPL-PEG-CH_3_ was 9.2 kDa (i.e., 4.1 kDa + 5.1 kDa). The M_w_ of the final, conjugated material was approximately 25.1 kDa after attachment of PLA (8.0 kDa)onto EPL-PEG-CH_3_. This value was 15.9kDa (≈ 8.0 × 2 kDa) higher than that of the original EPL-PEG-CH_3_.Correspondingly, materials containing 3 and 4 PLA grafted chains per EPL-PEG-CH3 molecule were obtained using EPL-PEG-CH_3_ to PLA feeding ratios of 1:5.7 (for PEP53) and 1:8.4 (for PEP54), respectively. These results verified that theEPL-PEG-CH_3_ to PLA feed ratio was the determining factor of the degree of PLA grafting.

### Structure of NPs

**Fig 2:**
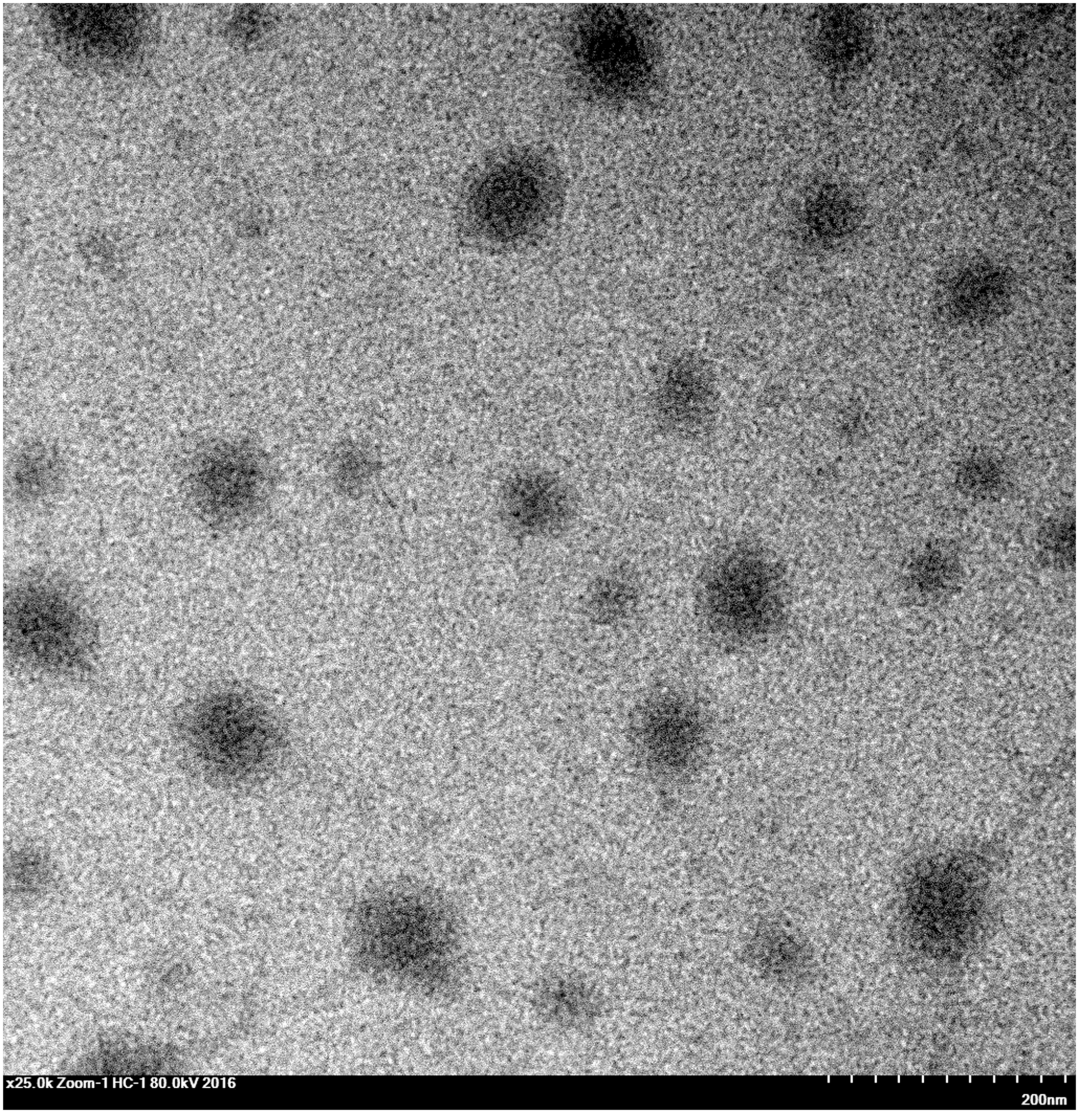
TEM image of PEP NPs.

The transmission electron micrographs in Fig 2 show PEP NPs with fine spherical shapes and aggregation. We used XPS to explore the nitrogen component percentage on the surface of blank NPs with different PEG M_w_ to observe whether the constructed carriers contained cores with PEG coating (Fig 1c).

We could detect just 2.68% nitrogen from the blank NPs (PEP02), which did not contain a PEG segment. This value significantly decreased with increasing M_w_ values of PEG. The XPS results detected no nitrogen for the products with PEG M_w_ higher than 5 kDa. These findings revealed that the PEG segments resided on the NP surface, with the PLA intertwining with EPL to form the NP core. Table 2 illustrates that the PEP NPs zeta potential was reduced from 48.62 to 21.17 mV with increasing PEG M_w_ (0–10 kDa), and the size was enlarged from 84.55 to 130.57 nm. The above findings confirmed that the PEG segments surrounded the core.

**Table 2:** WU, LC, zeta potential, and particle size of NPs.

^Δ^MNPs	Size (nm)	PDI	Zeta (mV)	LC (%)	WU (%)
PEP02	84.55 ± 3.57	0.09	48.62 ± 0.98	18.4	85.3
PEP12	90.28 ± 3.93	0.11	40.38±1.70	20.0	109.4
PEP22	98.73 ± 2.64	0.07	36.06 ± 0.64	24.4	110.8
PEP52	112.60 ± 4.50	0.11	31.07 ± 0.96	27.0	112.7
PEP53	103.21 ± 2.76	0.21	23.83 ± 1.39	22.7	119.4
PEP54	80.43 ± 4.78	0.28	18.48 ± 0.92	20.5	146.2
PEP102	130.57 ± 8.21	0.23	21.17 ± 2.33	29.8	135.1
PLA8	129.85 ± 2.54	0.17	−23.92 ±0.25	14.2	101.8

^Δ^MNPs denote the nanoparticles made of different materials. PDI is the polydispersity index of the NPs. LC is the BSA loading capacity in the NPs. WU is the water uptake of the corresponding nanoparticles at 2 days.

In order to further confirm whether PEP nanoparticles were coated with PEG shell, Lyophilized NPs were re-suspended in 1.0 mL D_2_O for ^1^H NMR analysis. In the NPs spectrum, PEG signals were clearly observed. However, the signal from PLA and EPL could not be seen. These results further suggested that PLA might intertwine with EPL blocks forming the core, and PEG segments formed the corona of the nanoparticles (Fig 1b).

### NP Water Absorption and Degradation

Degradation could be associated with many factors, such as crystallinity, hydrophilicity (hydrophobicity), porosity, and polymeric relaxation. However, hydrolysis is one of the most important factors in PEP NPs degradation, which is discussed in detail here.

The water uptake of the PEP52 NPs was 112.7%, which was higher than that of the PLA NPs (101.8%) (Table 2). This result might be attributed to the presence of hydrophilic PEG-EPL. The water absorption of the NPs increased from 110.8% to 135.1% with increasing PEG M_w_ (2 kDa (PEP22) to10 kDa (PEP102)) in PEP. High M_w_ PEG could absorb a large amount of water and eventually induce NP degradation.

For PEPs having identical PEG-EPL segments (e.g., PEP52, PEP53, and PEP54), the water absorption increased with increasing PLA grafting degree (from 112.7% to 146.2%). This phenomenon could be attributed to the possibility that NPs from copolymers with higher PLA grafting degrees have a loose structure, thereby facilitating the penetration of water molecules into the matrix of the polymer.

Fig 1d shows the weight reduction of samples incubated in PBS for 32 days, which illustrates an evident degradation. Gradual weight reduction in PLA was observed during the first eight days, which was attributed to the hydrophilic PEG-EPL segments within PEP. This characteristic might offer more pathways for water absorption and correspondingly lead to a faster hydrolysis rate than that of the PLA NPs. However, the weight of PLA NPs decreased faster than the other NPs after eight days. This phenomenon indicates that the acidic degradation products in the NPs induced an autocatalytic hydrolysis reaction within the polymer matrix, which further increased water uptake and degradation. Furthermore, the PEP NPs could inhibit the autocatalytic hydrolysis caused by the degradation of PLA, thus creating a mild microenvironment for the encapsulated proteins.

Lactic acid is produced during NP degradation by PLA hydrolysis. EPL can degrade to L-lysine or oligomers via hydrolysis in unstable aqueous lactic acid solutions [10]. The degradation products of EPL (i.e.,lysine or oligomers) can homogeneously pervade throughout the NPs to efficiently inhibit the autocatalytic degradation reactions. Here, NPs with moderate pH microclimates were produced by determining the appropriate PLA to PEG-EPL feeding ratio.

### Release and Kinetics of BSA *In Vitro*

**Fig 3:**
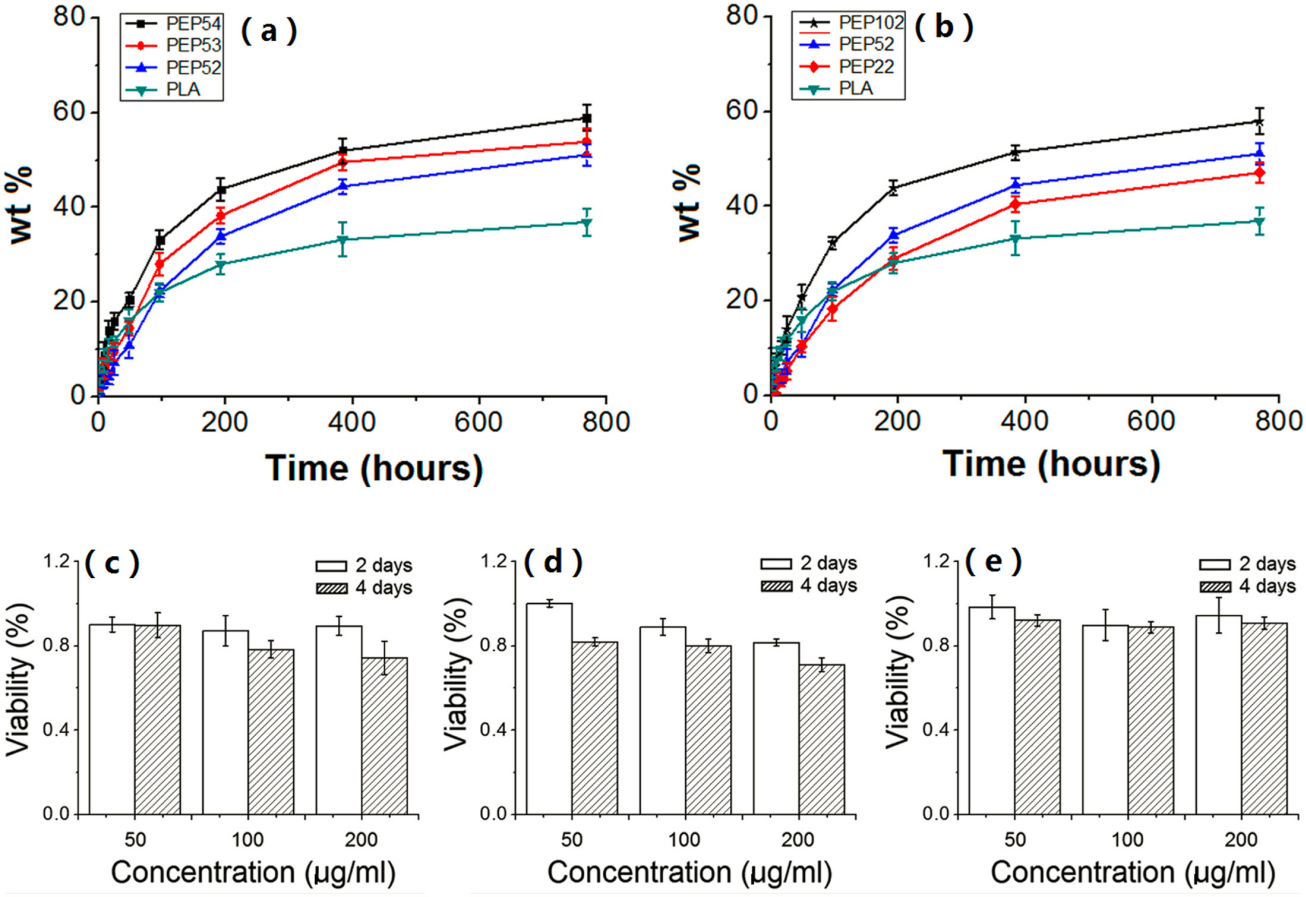
Protein release profiles and MTT assay of PEP NPs in the HL-7702 cell line. Protein release profiles of (a) PLA NPs, (b) PEP NPs, Cell viability result of (c) PLA NPs, (d) EPL, (e) PEP NPs. Cell viability was determined via MTT assay and expressed as a percentage of the control (100% of longitudinal coordinate).

BSA release overtime is illustrated in Fig 3. The curves showed a burst release following a slow continuous release phase. PLA NPs had an obvious initial burst release during the first 48 h. However, after 48 h, PLA NPs released a smaller amount of BSA during the subsequent 384 h period than any of the PEP NPs. This result may be attributed to the aggregation of BSA in the acidic microclimate within the PLA carriers, which is disadvantageous for BSA release.

Fig 3a displays the BSA release profiles from PEP NPs with different degrees of PLA grafting. The experimental data were first fitted to the Ritger–Peppas equation (Mt/Mn ≤ 60%), which is generally adopted to depict the release pattern from polymers if the mechanism is not known or if several types of release phenomena are considered [10]. Table 3 shows 0.43 < n < 0.85 for PEP35 and PEP25 NPs, thereby suggesting that the release mechanism was anomalous, which corresponds to coupled diffusion and polymer relaxation. By contrast, n ≤ 0.43 for PEP45 NPs, which indicated Fickian diffusion as the main mechanism of protein release.

**Table 3:** Kinetic fitting results of BSA released from nanoparticles with different kinetic models.

ΔΔΔMNPs	^Δ^Ritger–Peppas model			ΔΔFirst-order kinetic model				
	K_RP_	n	R^2^_adj_	a1	a2	k1	k2	R^2^_adj_
PEP54	5.058	0.382	0.971	52.787	46.251	0.014	0.000466	0.991
PEP53	3.310	0.435	0.958	49.632	47.960	0.007	0.000159	0.994
PEP102	4.289	0.407	0.958	52.037	46.673	0.013	0.000394	0.998
PEP52	1.981	0.503	0.956	47.051	52.471	0.006	0.000103	0.998
PEP22	1.505	0.531	0.966	46.236	53.437	0.005	0.000039	0.999
PLA	4.289	0.336	0.979	22.928	74.122	0.017	0.000220	0.983

Preparation condition: 10 mL BSA solution (0.4 mg/mL) was used as the aqueous phase, and other conditions were the same as those described in the paper.

^Δ^Ritger–Peppas model: M_t_/M_∞_ = K_RP_t^n^ (M_t_/M_∞_≤60%).

^ΔΔ^First-order kinetic model: M_t_/M_∞_ = 100−a_1_×exp(−k_1_t)−a_2_×exp(−k_2_t).

^ΔΔΔ^MNPs denote that nanoparticles made of different materials.

The initial BSA release from thePEP52 NPs was slower than those of PEP54 and PEP53 NPs. In the first 48 h, the percentage of released proteins from PEP52 NPs was merely 10.7%, while that of PEP54 NPs was 20.5%. The PEP54 NPs had the smallest average size and largest area-to-volume ratio, presumably facilitating the fast protein release on the surface. This phenomenon was verified via kinetic fitting (Table 3), in which the release amount (a1) and release rate (k1) of BSA increased with increasing PLA grafting degrees. The release profiles of the NPs after 48 h were similar. However, the release rate (k2) of BSA entrapped within the PEP54 NPs was the largest. This result may be explained by the more compact structure of PEP54 compared with those of PEP52 and PEP53. Such a structure induced a large steric hindrance between the PEP52 molecules during NP formation. Under such conditions, the matrix would have a looser structure, thereby providing numerous channels for water absorption and BSA release. Furthermore, the PEP54 NPs had the smallest average particle size, indicating a short diffusion path and thereby a fast release of entrapped proteins.

Fig 3b shows the BSA release profiles from PEP NPs with different PEG M_w_ (PEP22, PEP52, and PEP102). First, the initial burst release increased with increasing PEG M_w_. This increase may be due to the ability of long hydrophilic PEG segments to retain a large amount of BSA on the NP surface, resulting in a high BSA release rate. Second, the release rate of the protein entrapped in the NPs increased with an increasing number of PEG molecules. We speculated that a portion of PEG might have been embedded into the matrix during NP formation, thereby introducing channels for water uptake and BSA release.

The structural characteristics of the copolymer can significantly affect its release behavior. This result indicates that protein release can be manipulated by changing several basic structural properties, which is important for constructing a controlled release carrier of proteins with a specific release profile.

### Biocompatibility of PEP NPs

The positive charges of the NP carriers generally have detrimental effects on biocompatibility. In this work, all blank NPs exhibited positive zeta potentials (range: 18.48 ± 0.92 to 48.62 ± 0.98 mV) (Table 2). Therefore, the biocompatibility of the NPs should be verified. The possible toxicity of PEP NPs was examined by cytotoxicity and acute toxicity experiments.

Fig 3c–3e illustrates the outcome of an MTT assay using the HL-7702 cell line. On the fourth day, PLA NPs (200 mg/mL) and EPL groups presented cytotoxicity, with cell viability below 80%. By contrast, cell viability on the fourth day in the PEP52 NPs group was higher than 80%at the different concentrations tested. Increasing the NP concentration did not generate any obvious cytotoxicity. According to USP standards [15], the NPs were not cytotoxic. This finding can be interpreted as follows: the PEP NPs, which are composed of a PLA-EPL core with a hydrophilic PEG shell coating, contain a positively charged core that is coated with biocompatible PEG segments (Fig 1b), which may contribute to effectively decrease the cytotoxicity of PEP NPs.

**Fig 4:**
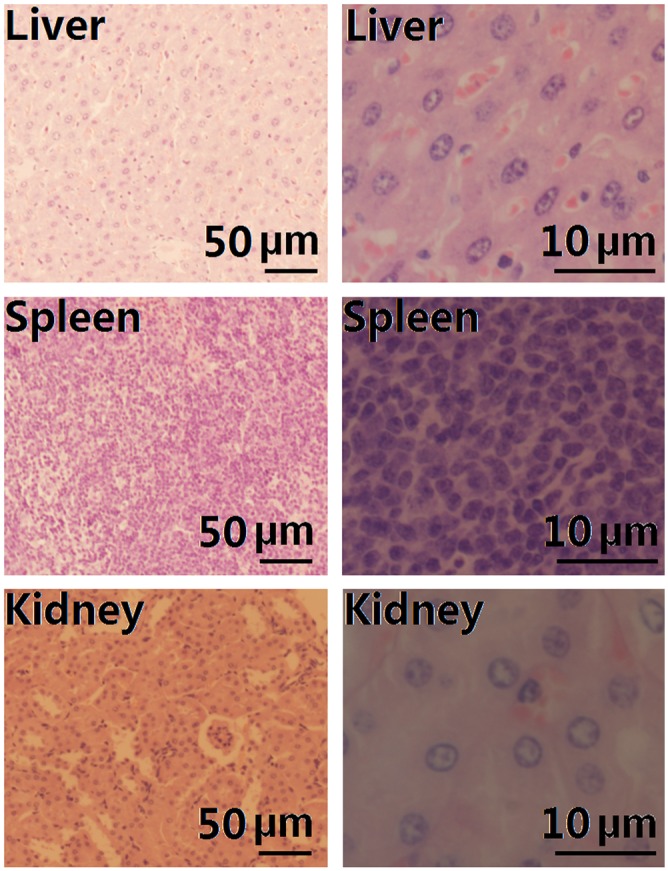
H&E-stained section of the liver, spleen, and kidney after injection of PEP52 NPs *in vivo*.

Our findings were also consistent with the results from the acute toxicity analysis of the PEP NPs (Table 4). Although no death was observed in the PLA NP group at the highest concentration of 540 mg/kg, the acute toxicity (LD 50) dose of EPL following tail vein injection was 272.6 mg/kg. No death was observed in the PEP NP group on day 14 at 2.160.0 mg/kg, the highest concentration of PEP NPs produced by nanoprecipitation. Thus, the LD 50 of the PEP NPs was determined to be the gram equivalent of 2,160.0 mg/kg, which is safe for administration [16, 17]. No abnormality was found in the livers, spleens, or kidneys of animals treated with PEP NPs after 14 days (Fig 4).

**Table 4:** Acute toxicity of PEP52 NPs, PLA NPs, and EPL.

Group	Number of mice	PEP285 NPs		PLA NPs		EPL solution	
		Doses (mg/kg)	Number of dead mice	Doses (mg/kg)	Number of dead mice	Doses (mg/kg)	Number of dead mice
1	10	Control	0	Control	0	Control	0
2	10	2160.0	0	540.0	0	191.0	0
3	10	**—**	**—**	**—**	**—**	227.3	2
4	10	**—**	**—**	**—**	**—**	270.5	5
5	10	**—**	**—**	**—**	**—**	321.9	7
6	10	**—**	**—**	**—**	**—**	383.0	9
7	10	**—**	**—**	**—**	**—**	450.0	10
LD50 (mg/kg)		**—**		**—**		280.4	

In the control group, each animal received sodium chloride solution (0.9%) via intravenous injection. The NPs were prepared as described above.

These results further implied that the PEP NPs were coated by biocompatible PEG segments, thereby contributing to effectively decrease the acute toxicity of the carriers.

### Mapping of Microclimate pH within PLA and PEP NPs

SNARF-1is a long-wavelength fluorescent pH indicator and a superior pH sensor. An acceptable pH range of pH 5.8 to 8.0 in could be established with image processing using this fluorescent indicator [18].We first used PBS (pH 5.8–8.0) to obtain a standard calibration curve of pHversusI_640_/I_580_and proceeded to examine the pH microenvironment within the NPs [10], the concentration of which was0.8 mg/mL.

**Fig 5:**
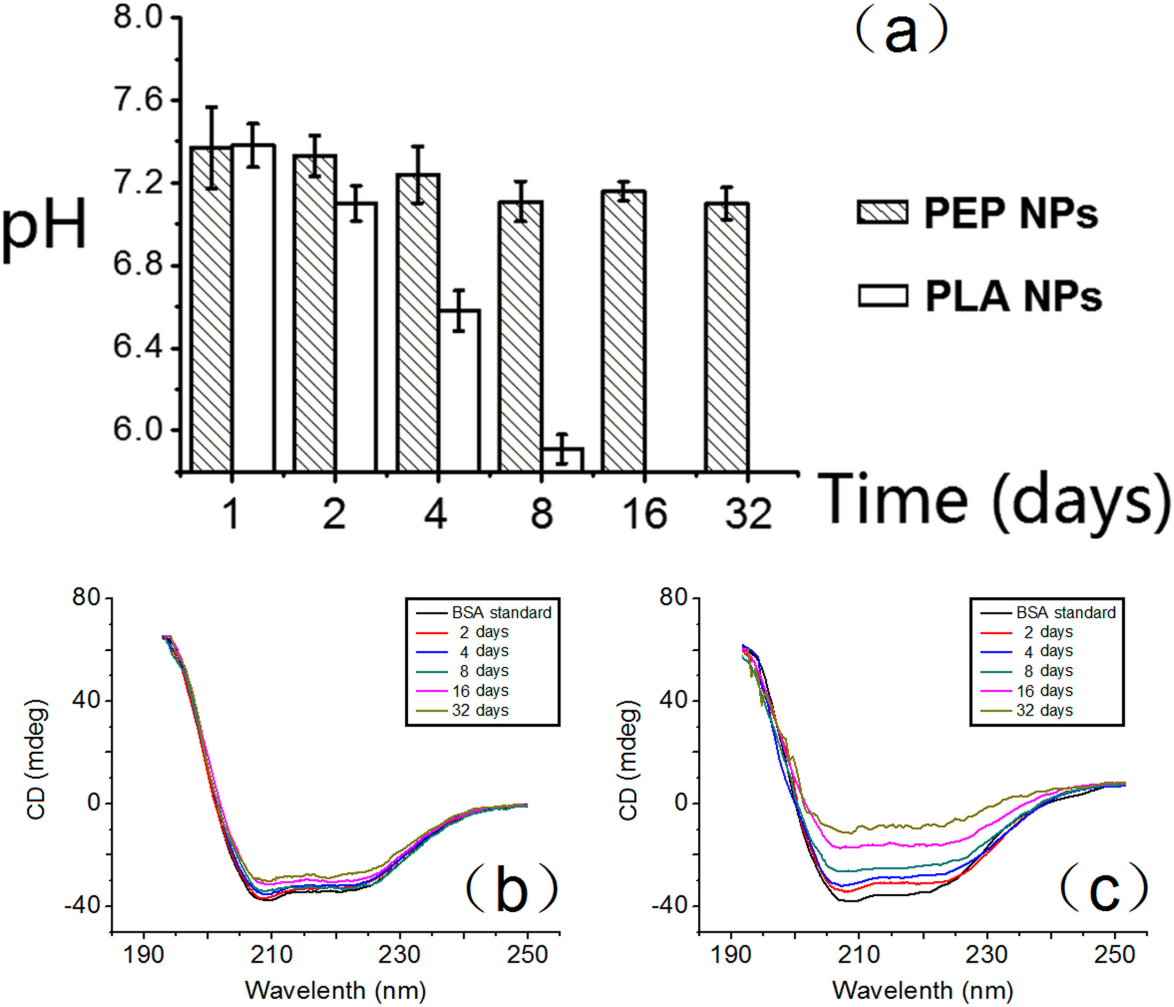
Microclimate pH of NPs and circular dichroism spectra of BSA. (a) Microclimate pH inside PLA and PEP52 NPs over 32 days. After the 16th day, the microclimate pH inside the PLA5 NPs was less than 5.8; (b) circular dichroism spectra of BSA released from PEP NPs and (c) PLA NPs over 32 days in PBS (0.1 M, pH 7.4).

Fig 5a shows the pH values withinPEP52 and PLA NPs. The microclimate pH levels of the PLA NPs decreased gradually over time because of the production of acidic degradation products. After 16 days, the pH level within the PLA NPs was reduced to pH<5.8. These findings suggested that hydrolysis of the ester bonds in PLA resulted in an acidic environment within the PLA NPs. Autocatalysis promoted by lactic acid produced many carboxylic acid materials inside the carriers, thereby increasing the acidity within the NPs. In contrast, the pH inside the PEP52 NPs remained relatively constant (pH range: 7.10–7.37) even after 32 days. These results indicated that the degradation of PLA did not induce an obvious reduction in pH inside the PEP52 NPs.

### Secondary Structural Stability of Released Proteins

We adopted CD spectroscopy to detect the denaturation of the released BSA from the NPs Fig 5b and 5c displays the features of the α-helix structure of standard BSA at 32 days. The estimated α-helicity was 60.5% according to molar ellipticity values for standard BSA. The release curve of BSA from PEP52 NPs on days 2, 4, 8, 16, and 32 showed obvious wave hollowing at 208 nm and 220 nm, with estimated helix contents of 58.3%, 57.9%, 56.7%, 52.4%, and 50.8%, respectively. In contrast, the protein released from PLA NPs after 32 days presented lower α-helix contents compared with that of the proteins released from PEP52 NPs. The estimated α-helix contents on days 2, 4, 8, 16, and 32 were 54.1%, 51.6%, 42.5%, 36.7%, and 30.8%, respectively. Hence, the PEP52 NPs more effectively protected BSA from inactivation compared with PLA NPs, especially after 16 days. This result could be attributed to the following: proteins might have been inactivated due to unfolding, aggregation, or damaged peptide bonds caused by the acidic products of PLA degradation. The comparatively alkalescent EPL in the PEP52 NPs effectively suppressed any significant pH decrease inside the NPs.

### Distribution and Pharmacokinetics of NPs

**Fig 6:**
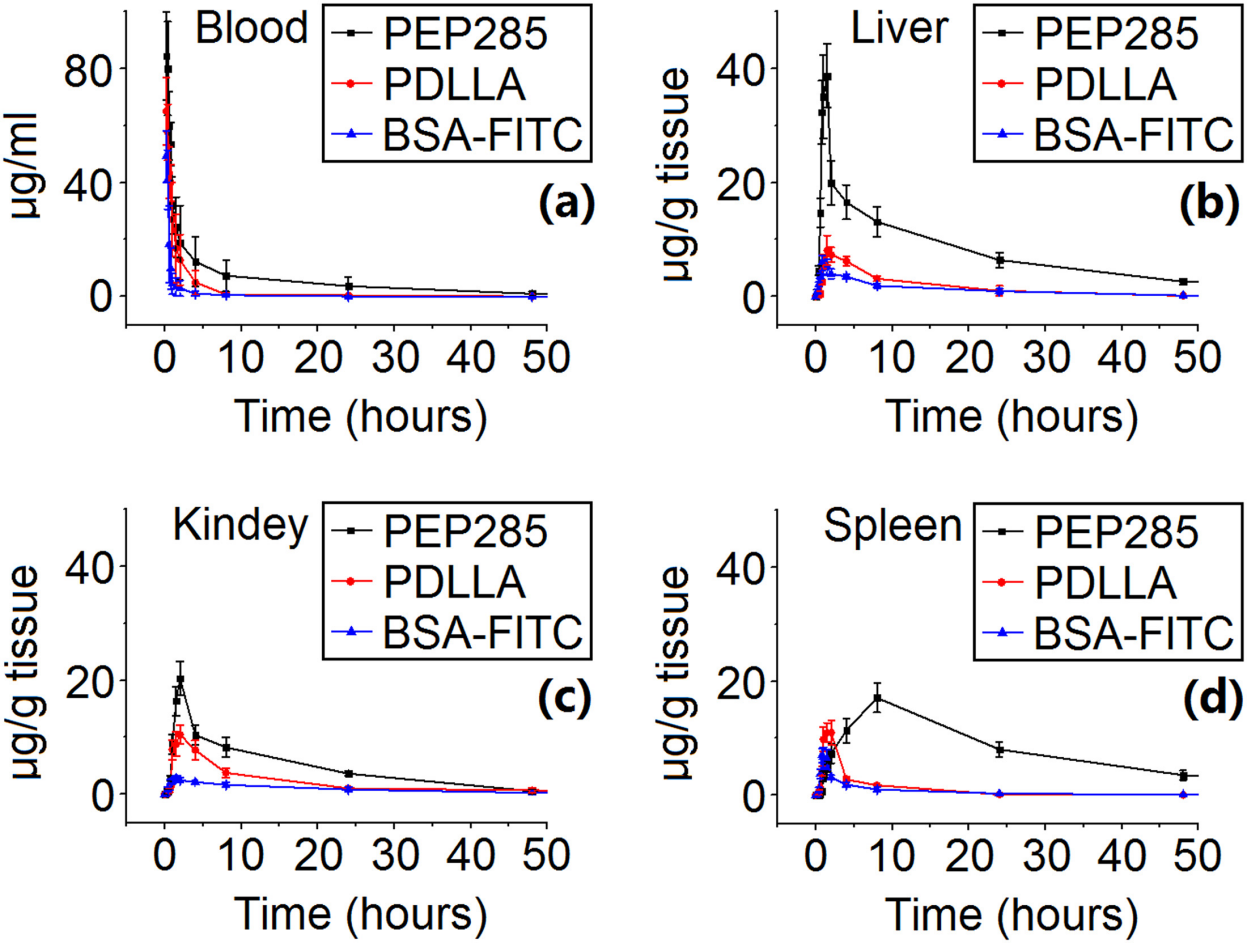
Time course of BSA-FITC concentration in plasma (a) and tissues [liver (b), spleen (c), and kidney (d)] over 16 days after intravenous injection of free BSA-FITC solution and different BSA-FITC-loaded carriers.

Fig 6 illustrates the distribution of BSA-FITC and NPs loaded with BSA-FITC in Kunming mice after intravenous injection. Fig 6 shows the average fluorescence distribution in the blood and organs (e.g., livers, spleens, and kidneys) at 16 days after various BSA-FITC treatments as shown in S2 Fig. We adopted the trapezoidal rule [14] to evaluate the area under BSA-FITC curves between *t*_0_ and *t* and computed AUC_t0–t_values.

The fluorescence levels in the blood and organs of the mice treated with PEP52 NPs were significantly higher than those obtained after the administration of PLA NPs, EP02 (ε-polylysine-g-PLA) NPs, and BSA-FITC (*P* < 0.05). The serum levels of BSA-FITC and NPs loaded with the former showed a rapid decrease with time, manifesting single exponential decay curves (Fig 6). These results demonstrated that PEP52 NPs circulated in the blood longer than PLA NPs, EP02 NPs, or BSA-FITC alone. Comparison of the AUCs between PEP NPs and BSA-FITC showed that the fluorescence levels of PEG-modified NPs were found to be significantly higher than those of the control and those obtained after PLA and EP02 NP treatments (*P*< 0.05). These findings suggest that PEGylated NPs exhibited a favorably longer circulation time than other carriers, including those with BSA-FITC.

Table 5 shows that the AUC_0–384 h_ values of the PEP52 NPs in the liver, spleen, and kidney were 1136.978, 986.809, and 603.238 μg/(g tissue), respectively. This finding indicates that the PEP52 NPs were cleared in the order of the liver, spleen, and kidney.

**Table 5:** AUC values of BSA-FITC-loaded formations and free BSA-FITC in the tissues and blood of Kunming mice (n = 10).

	Tissue	PEP52	PLA	BSA-FITC
AUC_0–384 h_ (μg/g tissue)	Liver	1136.978	286.769	169.094
	Spleen	986.809	116.686	101.343
	Kidney	603.238	363.835	83.238
AUC_0.2–384 h_ (μg/mL)	Blood	328.738	110.061	36.668

## Conclusions

We have determined that PEP NPs could maintain a suitable pH microclimate for continuous BSA release. In our work, a suitable pH level could be achieved within the NPs by either adjusting the degree of PLA grafting onto PEG-EPL, or adjusting the M_w_ of the two products. The various structural features of the protein delivery carriers could be adjusted to meet specific release requirements. Furthermore, PEP NPs showed satisfactory biocompatibility and circulation times. Hence, the PEP NPs are suitable carriers for continuous release of bioactive proteins.

## Supporting Information

S1 Fig ^1^H-NMR spectrum of PEP in DMSO-d6. (TIF)

S2 Fig Time course of BSA-FITC concentration in plasma (a) and tissues [liver (b), spleen (c) and kidney (d)] for 16 days after intravenous injection of free BSA-FITC solution and BSA-FITC loaded carriers. (TIF)

S1 File Detailed description of PEP preparation and characterization. The distribution and pharmacokinetics of PEP NPs.
(DOC)
