# Supplementary figures and images for: pH-Responsive Polyethylene Glycol Monomethyl Ether-ε-Polylysine-G-Poly (Lactic Acid)-Based Nanoparticles as Protein Delivery Systems
Source: PLoS One. 2016 Jul 28;11(7):e0159296. doi: 10.1371/journal.pone.0159296 (PMC4964987; doi:10.1371/journal.pone.0159296)

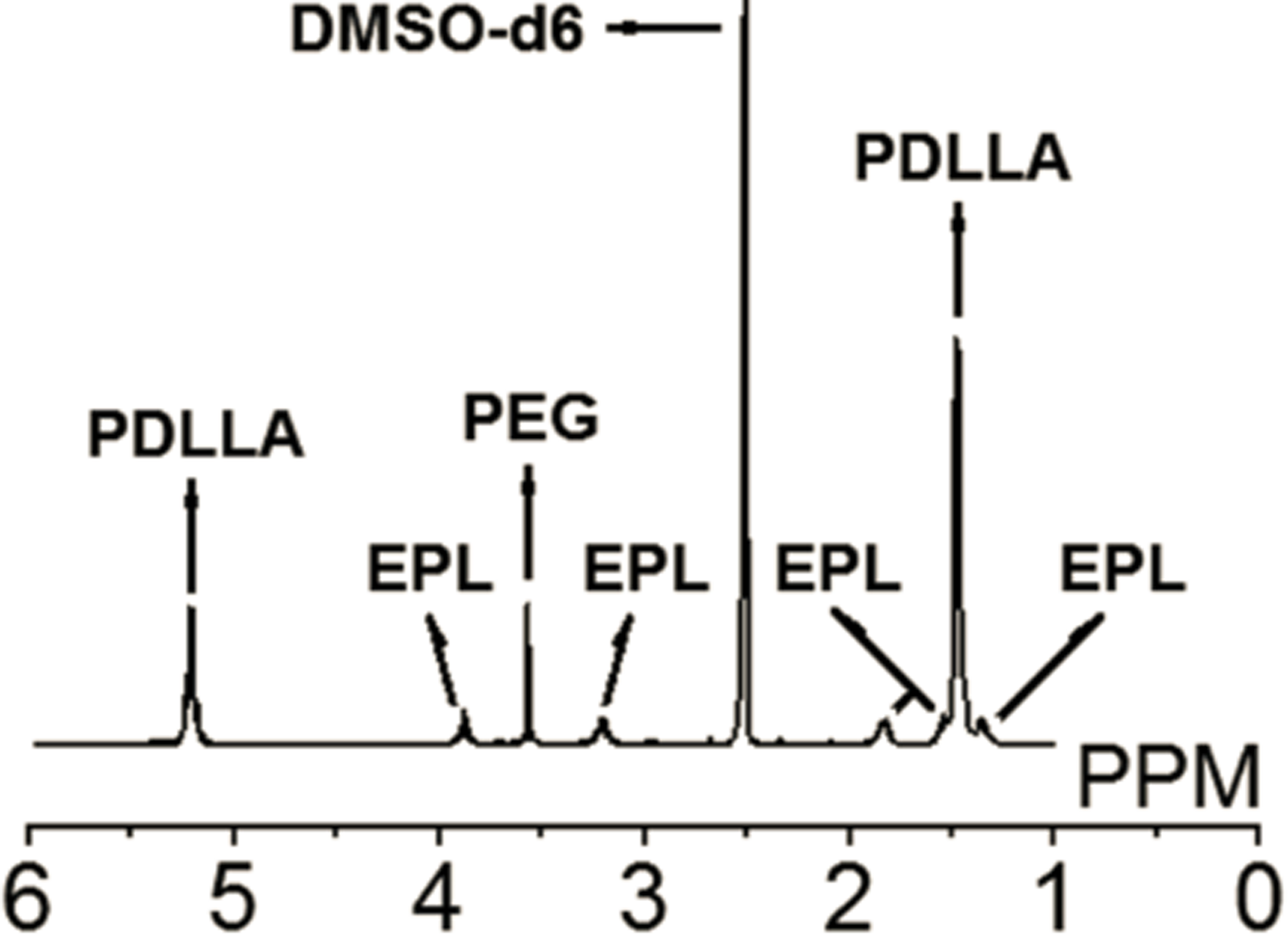

Supplement: S1 Fig — (TIF) [file pone.0159296.s001.tif]

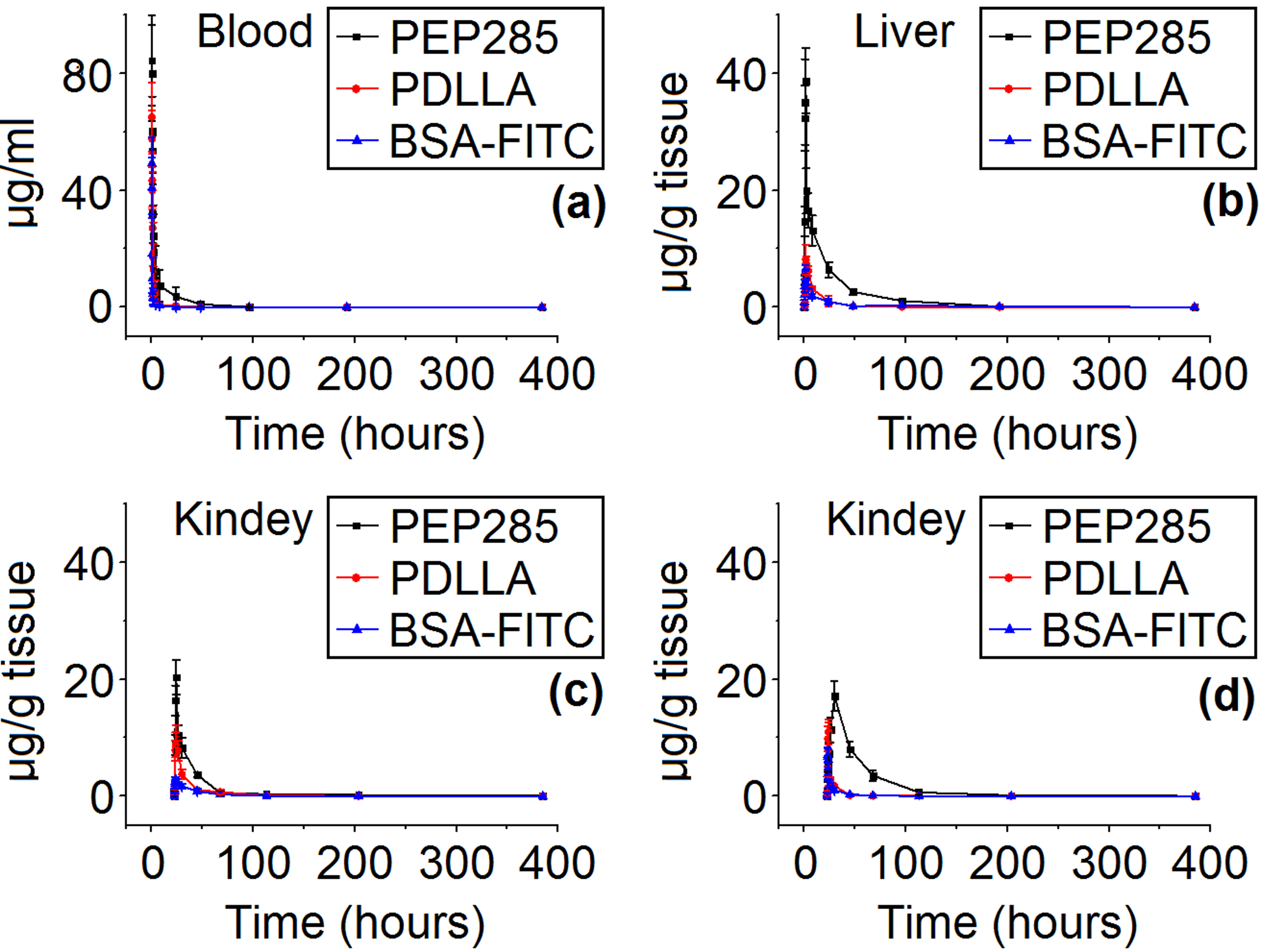

Supplement: S2 Fig — (TIF) [file pone.0159296.s002.tif]
