# Supplementary material for: pH-Responsive Polyethylene Glycol Monomethyl Ether-ε-Polylysine-G-Poly (Lactic Acid)-Based Nanoparticles as Protein Delivery Systems
Source: PLoS One. 2016 Jul 28;11(7):e0159296. doi: 10.1371/journal.pone.0159296 (PMC4964987; doi:10.1371/journal.pone.0159296)
Supplement: S1 File — The distribution and pharmacokinetics of PEP NPs. (DOC) [file pone.0159296.s003.doc]

**Supporting Information to:**

pH-responsive polyethylene glycol monomethyl ether-ε-polylysine-g-poly (lactic acid)-based nanoparticles as protein delivery systems

Hiuqin Liu^1^, Yijia Li^2^, Ning Zhang^1,3^, Xiujun Gao^2,3^*, and Guoguang Ying^1^*

^1^Laboratory of Cancer Cell Biology, Tianjin Key Laboratory of Cancer Prevention and Therapy, National Clinical Research Center for Cancer, Tianjin Medical University Cancer Institute and Hospital, Tianjin 300060, China

^2^Institute of Biomedical Engineering, Tianjin Medical University, Tianjin 300070, China

^3^Research Center of Basic Medical Sciences, Tianjin Medical University, Tianjin 300070, China

*Corresponding author:

Xiujun Gao, E-mail: [sciputonsteam@163.com](mailto:sciputonsteam@163.com)；Tel/Fax: +86-22-83336939

Guoguang Ying, E-mail: [lhq2335@126.com](mailto:lhq2335@126.com)；Tel/Fax: +86-22-23522919

**PEP preparation & characterization**

Primary amines of EPL were protected by Di-tert-butyl dicarbonate (BOC) groups. Briefly, 2 mmol of EPL were suspended in ethanol/water (15 ml) and 3 mmol of BOC were added to the solution while stirring at room temperature under a nitrogen atmosphere. After 6 hours, the mixture was precipitated by centrifugation. The product was washed 3 times with ethanol, and dried in vacuo for 48 h to give the BOC-protected EPL (BOCEPL). ^1^H NMR (DMSO-d6): δ 1.39 (CH_3_ of BOC); 1.40-1.89 (CδH_2_-CγH_2_-CβH_2_ of EPL, 6H), 3.25 (CεH_2_ of EPL, 2H), 3.94 (CαH of EPL, 1H).

The reaction of the primary amine of H_2_N-PEG-CH_3_ with the terminal carboxyl group of BOCEPL was catalysed by EDC·HCl and HOBt as coupling agents. EDC·HCl (2 mmol) in DMF (5 ml) was poured into a solution of BOCEPL (1 mmol) and HOBt (2 mmol) in DMF (15 ml) with one drop of triethylamine (TEA). The solution was stirred at room temperature for 0.5 h and allowed to be added dropwise to H_2_N-PEG-CH_3_ (1.5 mmol) in 30 ml DMF at 45 ℃. After 3 days under N_2_, the product was poured into 200 ml of cold diethyl ether under vigorous stirring. The precipitate was filtered out and washed three times with ethanol and water, respectively, and dried in vacuo for 48 h to give the BOCEPL-PEG-CH_3_. ^1^H NMR (DMSO-d6): δ 3.12 (C_a_H_2_-N of PEG, 2H), 3.59 (CH_2_-CH_2_-O of PEG, 4H), 3.31 (CH_3_-O of PEG, 3H); the other ppm values of the product were described above.

Deprotection of BOC groups from BOCEPL-PEG-CH_3_ was achieved by trifluoroacetic acid (TFA). Briefly, the BOCEPL-PEG-CH_3_ was dissolved in DMF, and then an excess amount of TFA was added to the organic solution to remove the protecting BOC groups. The product was precipitated by cold aether and dried in vacuo at 50 ℃ to give EPL-PEG-CH_3_. The ppm values in ^1^H NMR spectrum of the product (EPL-PEG-CH_3_) were described above.

A typical coupling reaction procedure of PLA with EPL-PEG-CH_3_ was as follows: EDC·HCl in DMF (20 ml) was poured into a solution of PLA and HOBt in DMF (50 ml) with one drop of triethylamine (TEA). The solution was stirred at room temperature for 0.5 h and allowed to be added dropwise to EPL-PEG-CH_3_ (1 mmol) in DMF at 45 ℃. After 3 days under N_2_, the product was poured into 200 mL of cold diethyl ether under vigorous stirring. The precipitate was filtered out and washed with water and methylene chloride three times to remove small impurities and PLA, respectively (The product was able to dissolve in DMF, but could not dissolve in methylene chloride). The filtrate, a yellow powder, was then acquired by vacuum drying as the final production (PEP).

It is noteworthy that the coupling reaction should be performed at a lower temperature (below 45 ℃) and under nitrogen atmosphere because oxidation of amine groups took place on exposure to air at high temperature.

The number of PLA chains grafted onto EPL-PEG-CH_3_ increased as the feeding ratio of PLA to EPL-PEG-CH_3_ increased as shown in Table 1. In addition, with a similar EPL-PEG-CH_3_/PLA ratio, the graft degree of PLA decreased as its molecular mass increased. The obtained results indicated that the steric hindrance of the association of PLA with EPL-PEG-CH_3_ backbone might increase the approaching resistance between EPL-PEG-CH_3_ and the other free PLA. During the same reaction time, the grafting degrees of PLA could be controlled by the feeding ratio of PLA to EPL-PEG-CH_3_, which is a very important factor to stabilize protein and adjust release rate of the carriers.

Figure 1 in S1 File: ^1^H-NMR spectrum of PEP in DMSO-d6.

The ^1^H NMR spectrum of the copolymer PEP is demonstrated in the figure above.

The molecular weights and structures of the copolymers were further confirmed by GPC. The products were much pure because only a single peak was appeared and the polydispersity index of molecular weights was small in the GPC measurement (the spectra were not shown).

**Distribution and pharmacokinetics of NPs**

The distribution profiles of free BSA-FITC and carriers loaded with BSA-FITC in Kunming mice for 384 hours after intravenous administration were shown in the following figure.

Figure 2 in S1 File: Time course of BSA-FITC concentration in plasma (a) and tissues [liver (b), spleen (c) and kidney (d)] for 16 days after intravenous injection of free BSA-FITC solution and BSA-FITC loaded carriers.
